# *ARID5B*, *IKZF1* and Non-Genetic Factors in the Etiology of Childhood Acute Lymphoblastic Leukemia: The ESCALE Study

**DOI:** 10.1371/journal.pone.0121348

**Published:** 2015-03-25

**Authors:** Jérémie Rudant, Laurent Orsi, Audrey Bonaventure, Stéphanie Goujon-Bellec, André Baruchel, Arnaud Petit, Yves Bertrand, Brigitte Nelken, Marlène Pasquet, Gérard Michel, Laure Saumet, Pascal Chastagner, Stéphane Ducassou, Yves Réguerre, Denis Hémon, Jacqueline Clavel

**Affiliations:** 1 Institut national de la santé et de la recherche médicale (INSERM) U1153, Epidemiology and Biostatistics Sorbonne Paris Cité Center (CRESS), Epidemiology of childhood and adolescent cancers team (EPICEA), Villejuif, France; 2 Paris-Descartes University, UMRS-1153, Epidemiology and Biostatistics Sorbonne Paris Cité Center (CRESS), Paris, France; 3 French National Registry of Childhood Hematopoietic Malignancies (RNHE), Villejuif, France; 4 Assistance Publique-Hôpitaux de Paris, Hôpital Robert Debré, Paris, France; 5 Université Paris Diderot-Paris 7, Sorbonne Paris Cité, Paris, France; 6 Assistance Publique-Hôpitaux de Paris, HUEP, Hôpital Trousseau, Paris, France; 7 Sorbonne Universités, UPMC Univ Paris 06, UMR S_938, Paris, France; 8 Institut d’Hémato-Oncologie Pédiatrique, Lyon, France; 9 Université Claude Bernard Lyon 1, Lyon, France; 10 Pôle Enfant, CHRU, Lille, France; 11 Université Lille Nord de France, Lille, France; 12 Hématologie pédiatrique, CHU Toulouse, INSERM U1037, Equipe 16, CRCT, Oncopôle, Toulouse, France; 13 Department of Pediatric Hematology and Oncology, Timone Enfants Hospital, Marseille, France; 14 Aix-Marseille University, Marseille, France; 15 Hôpital Arnaud de Villeneuve, Montpellier, France; 16 Hôpital d’enfants, CHU de Nancy, Vandoeuvre, France; 17 Hôpital Pellegrin Tripode, Bordeaux, France; 18 CHU Félix Guyon, Saint Denis de La Réunion, France; National Health Research Institutes, TAIWAN

## Abstract

Genome-wide association studies (GWAS) have identified that frequent polymorphisms in *ARID5B* and *IKZF1*, two genes involved in lymphoid differentiation, increase the risk of childhood acute lymphoblastic leukemia (ALL). These findings markedly modified the current field of research on the etiology of ALL. In this new context, the present exploratory study investigated the possible interactions between these at-risk alleles and the non-genetic suspected ALL risk factors that were of sufficient prevalence in the French ESCALE study: maternal use of home insecticides during pregnancy, preconception paternal smoking, and some proxies for early immune modulation, i.e. breastfeeding, history of common infections before age one year, and birth order. The analyses were based on 434 ALL cases and 442 controls of European origin, drawn from the nationwide population-based case-control study ESCALE. Information on non-genetic factors was obtained by standardized telephone interview. Interactions between rs10740055 in *ARID5B* or rs4132601 in *IKZF1* and each of the suspected non-genetic factors were tested, with the SNPs coded as counts of minor alleles (trend variable). Statistical interactions were observed between rs4132601 and maternal insecticide use (p = 0.012), breastfeeding p = 0.017) and repeated early common infections (p = 0.0070), with allelic odds ratios (OR) which were only increased among the children not exposed to insecticides (OR = 1.8, 95%CI: 1.3, 2.4), those who had been breastfed (OR = 1.8, 95%CI: 1.3, 2.5) and those who had had repeated early common infections (OR = 2.4, 95%CI: 1.5, 3.8). The allelic ORs were close to one among children exposed to insecticides, who had not been breastfed and who had had no or few common infections. Repeated early common infections interacted with rs10740055 (p = 0.018) in the case-only design. Further studies are needed to evaluate whether these observations of a modification of the effect of the at-risk alleles by non-genetic factors are chance findings or reflect true underlying mechanisms.

## Introduction

Acute lymphoblastic leukemia [ALL] accounts for 80% of all childhood leukemia and for approximately one quarter of all childhood neoplasms in developed countries [1]. Until recently, only Down’s syndrome, a few inheritable predisposing diseases and high-dose ionizing radiation were known to increase the risk of ALL [2,3]. Genome-wide association studies (GWAS) have revealed common Single Nucleotide Polymorphisms (SNP) associated with childhood ALL risk [4–12]. The two loci associated with the greater ALL risk are in the AT-rich interactive domain 5b gene (*ARID5B*, chromosomal region 10q21.2) and Ikaros family zinc finger 1 gene (*IKZF1*, chromosomal region 7p12.21). *IKZF1* codes for a zinc finger transcription factor, Ikaros, which is a key regulator of hematopoiesis. In particular, Ikaros is required for the development of the earliest B-cell progenitors and at later stages for V(D)J recombination and B-cell receptor expression [13,14]. Ikaros also acts as a tumor suppressor [15] and genetic alteration of *IKZF1* in leukemia cells is associated with a poor outcome in B-cell-progenitor ALL [16,17]. *ARID5B* is a member of the AT-rich interaction domain family of transcription factors that plays an important role in embryogenesis and growth regulation [18]. The potential role of *ARID5B* in immune response and lymphoid lineage differentiation has been less investigated than for *IKZF1*. A role is supported by data from homozygous knockout mice that had immune abnormalities and reduced numbers of B-cell progenitors [19].

The discovery of some ALL susceptibility loci calls for revisiting the suspected environmental risk factors for childhood ALL in light of the possible interactions those factors may have with the variant alleles of *ARID5B* and *IKZF1*. The relationships between ALL and the exposures may vary depending on *ARID5B* and *IKZF1* polymorphisms and, conversely, the genetic effects may be modified by environmental exposures. Indeed, growing evidence suggests that environmental factors may cause diseases by modifying the expression of genes involved in key cell functions, potentially through epigenetic deregulation [20–27]. Modeling their combined effect may enable insights into the underlying biologic processes and the potential implication of the environmental factors in ALL risk [28]. Several exposures are candidate risk factors for childhood ALL: pesticides, low dose ionizing radiation, extremely low frequency electromagnetic fields, benzene exposure, paternal smoking, absence of folic acid supplementation, infections transmitted through population mixing, a lack of early exposure to infectious agents, and absence of breastfeeding [2,3].

The exploratory study reported herein investigated the potential interactions between polymorphisms rs10740055 of *ARID5B*, and rs4132601 of *IKZF1*, and the non-genetic suspected ALL risk factors that were of sufficient prevalence in the ESCALE case-control study, for power reasons: maternal use of home insecticides during pregnancy, preconception paternal smoking, and some proxies for early immune modulation, i.e breastfeeding, history of repeated common infections before age one year, and birth order [29–31].

## Materials and Methods

### Case and control ascertainment

The ESCALE study is a population-based case-control study designed to assess the role of several environmental and genetic factors in childhood cancer, including ALL [29–31]. The ALL cases were children diagnosed with ALL between January 1, 2003, and December 31, 2004, as defined by the registration criteria of the National registry of childhood hematopoietic malignancies (RNHE). To be eligible, children were to be less than 15 years old and residing in France at the time of diagnosis. Children were not eligible if they had been adopted, or if their biological mother had died (n = 8), did not speak French (n = 19), had a serious psychiatric disorder (n = 12), or could not be interviewed for ethical reason because the child was in palliative care (n = 2) or had died (n = 17). Among the 714 eligible ALL cases, 648 cases (91%) had participated in the ESCALE study. Information on leukemia subtype and cytogenetic characteristics was subsequently obtained from the RNHE information system.

The population controls, children free from cancer, were contemporaneously selected in 2003–2004, using a quota sampling method. A base of 60,000 phone numbers representative of telephone subscribers was randomly extracted from the telephone directory (S1 Fig.). Then, controls were recruited using gender and age quotas in sixteen strata (boys and girls from the age strata 0–1, 2, 3, 4, 5–6, 7–8, 9–11 and 12–14 years) reflecting the expected distribution of all the cancer cases included in the ESCALE study, based on rates from the RNHE [32] and the Regional Childhood Cancer Registries [33]. Additional quotas were applied in order to make the number of children less than 15 years old living in the household similar to that of the French population, conditionally on age. Children who had been adopted, or whose biological mother had died, did not speak French, or had a serious psychiatric disorder were not eligible. Finally, 1681 of the 2360 eligible controls (71%) were enrolled in the ESCALE study.

A specimen was requested from each subject, and consisted in a blood sample for the cases, and a saliva sample collected using a swab brush for the controls. After obtaining parental consent, biological samples were taken from 619 (96%) of the 648 included ALL cases and 810 (48%) of the 1681 included controls, and 513 ALL cases and 570 controls had sufficient DNA for genotyping (S1 Table).

### Data collection

The mothers were interviewed by telephone, using the same standardized questionnaire for cases and controls. The mothers were asked for socio-demographic information, child and family medical history, and details on the child’s environment and lifestyle, birth characteristics and infancy care. The mothers were also asked whether they had used products against insects at home during pregnancy [30], if the father had ever smoked and the year he quitted [29], if their child had been breastfed, and if the child had had none, 1 to 3, or four or more infectious episodes for each of the following sites before age one year: tonsillitis, otitis, upper respiratory tract infections, gastroenteritis, bronchiolitis and other lower respiratory tract infections, and urinary tract infections [31]. A history of repeated early common infections was defined as 4 or more episodes of infection of at least one given site or 1–3 episodes of infection of at least 4 sites in infancy [31].

### Genotyping

The cases were genotyped on Illumina 370K-Quad BeadChips and the controls for 4,868 candidate SNPs on Illumina Infinium iSelect custom Beadchips (IntegraGen). Both beadchips included SNPs from at-risk loci revealed in the first GWAS on ALL, in *ARID5B* and *IKZF1* [4,5]. Quality control excluded 109 controls with an individual call rate less than 95% and 36 ALL cases with an individual call rate less than 97%, low or high heterozygosity or discrepancies between stated gender and sexual chromosomes [6]. Six cases were excluded because they had Down’s syndrome. Mean individual call rates were 99.9% for the cases and 98.5% for the controls. The SNPs of interest did not deviate from the Hardy–Weinberg equilibrium in the control sample.

The analyses were restricted to the 434 cases and 442 controls who had at least two European-born grandparents, given that this criterion proved to be a good proxy to identify children of European-descent in the present study [6]. The cases consisted in 10 pro-B ALL, 355 common B-cell ALL, 19 mature B-cell ALL, 40 T-cell ALL and 10 unspecified ALL (S1 Table). Eighty-two common B-cell ALL cases were ETV6-RUNX positive and 157 were hyperdiploid (≥ 47 chromosomes).

### Statistical analysis

The present analysis focused on rs4132601 for *IKZF1* and, on rs10740055 for *ARID5B*, as these SNPs were the most significantly associated with ALL in the ESCALE genotyped sample [6]. We selected the suspected non-genetic factors associated with ALL in our previous analyses, and to which at least 30% of the control population were exposed. The factors included paternal smoking since the year prior to the child’s birth [29], breastfeeding, repeated early common infections and birth order (2 and more versus 1) [31], and maternal use of home insecticides during pregnancy which was the pesticide exposure the most associated with acute leukemia in the ESCALE study [30]. The analyses addressing common infections were restricted to the children aged one year or more (398 controls and 426 ALL).

Odds ratios (OR) and their 95% confidence intervals (CI) were estimated using unconditional logistic regression models including the child’s age and gender, and parental socio-professional category. ORs were calculated for each stratum of the variables combining each polymorphism with each of the 5 non-genetic factors of interest. Interaction odds ratios (IOR) between the polymorphism and the exposure of interest were estimated by both case-control and case-only analyses, with the SNPs coded as counts of minor alleles (trend variable), and tested under the multiplicative model. The IORs that could be detected with a power of 80% and a two-tailed 5% level of significance were equal to 1.5 and 1.8 for the case-only and the case-control design, respectively, assuming a minor allele frequency of 30%, an allelic OR of 1.5, an exposure prevalence of 30% and an OR of association between ALL and the exposure of 1.5.

The research was conducted in accordance with the principles of the Declaration of Helsinki (World Medical Association, 2004) and complied with all applicable international regulatory requirements. In particular, the study was reviewed and approved by the French National Institute of Health and Medical Research ethics committee and the Direction Générale de la Santé institutional review board before the study began (DGS No. 2003/0259). Written informed consent was obtained from the parents of the children enrolled in the study.

All regression analyses were conducted in SAS version 9.3 and minimal detectable IORs were estimated using Quanto version 1.2.4 [34].

## Results

Table 1 reports the associations previously observed between childhood ALL and the polymorphisms rs4132601 in *IKZF1* and rs10740055 in *ARID5B* in the ESCALE study. The table also shows the associations with maternal use of home insecticides during pregnancy, preconception paternal smoking, birth order, breastfeeding and repeated early common infections observed both in the genotyped subsample used for the present analysis and in the whole ESCALE sample [29–31], with similar results.

**Table 1:** Association between childhood acute lymphoblastic leukemia (ALL), rs10740055 *(ARID5B)*, rs4132601 *(IKZF1)*, and the non-genetic factors of interest, in the ESCALE study, France 2003–2004.

	ESCALE sample of genotyped European-descent children				Whole ESCALE sample (1681 controls and 648 ALL)		
	Controls	ALL					Reference
	N = 442	N = 434	**OR** ^a^	95%CI	**OR** ^a^	95%CI	
***ARID5B*** (rs10740055)							[6]
AA	106	67		Ref.			
AC *vs*. AA	218	182	**1.4**	0.9, 2.0			
CC *vs*. AA	118	185	**2.5**	1.7, 3.7***			
C allele (allelic OR)	MAF = 0.51	MAF = 0.64	**1.6**	1.3, 1.9***			
***IKZF1*** (rs4132601)							[6]
TT	211	175		Ref.			
TG vs. TT	167	202	**1.4**	1.0, 1.8			
GG vs. TT	37	57	**1.9**	1.1, 3.0*			
missing	27	0					
G allele (allelic OR)	MAF = 0.29	MAF = 0.36	**1.4**	1.1, 1.7**			
**Maternal use of home insecticides during pregnancy**							[30]
No	283	222		Ref.		Ref.	
Yes	149	185	**1.6**	1.2, 2.2***	**1.7**	1.4, 2.1***	
missing	10	27					
**Preconception paternal smoking**							[29]
No	238	191		Ref.		Ref.	
Yes	196	229	**1.3**	1.0, 1.7	**1.4**	1.1, 1.7*	
missing	8	14					
**Breastfeeding**							[31]
No	191	225		Ref.		Ref.	
Yes	251	209	**0.8**	0.6, 1.1	**1.0**	0.8, 1.2	
**Repeated early common infections** ^b^							
No	246	288		Ref.		Ref.	[31]
Yes	127	123	**0.8**	0.6, 1.1	**0.8**	0.6, 0.9*	
missing	25	15					
**Birth order**							[31]
First born	181	223		Ref.		Ref.	
Second born and over	261	211	**0.5**	0.4, 0.7**	**0.7**	0.6, 0.8**	

ALL: acute lymphoblastic leukemia; CI: confidence interval; MAF: minor allele frequency; OR: odds ratio; SNP: single nucleotide polymorphism

*:p<0.05;

**: p<0.01;

***: p<0.001

^a^ OR and 95%CI estimated by unconditional logistic models including age and gender, and for non-genetic factors, parental socio-professional category.

^b^ Restriction to children ≥ 1 year old

The combined variables and the joint effects are shown in Table 2.

**Table 2:** Joint associations between childhood acute lymphoblastic leukemia (ALL) and the variables combining the non-genetic factors of interest with rs10740055 *(ARID5B)* and rs4132601 *(IKZF1)* polymorphisms. The analyses addressing common infections were restricted to the children aged one year or more (398 controls and 426 ALL), while the analyses addressing the other exposures were based on the whole sample (442 controls and 434 ALL).

**rs10740055 (*ARID5B*)**							**rs4132601 *(IKZF1)***						
	Co/ALL	**OR** ^**a**^	95%CI	Co/ALL	**OR** ^**a**^	95%CI		Co/ALL	**OR** ^**a**^	95%CI	Co/ALL	**OR** ^**a**^	95%CI
	**Maternal use of home insecticides during pregnancy**							**Maternal use of home insecticides during pregnancy**					
		No			Yes				No			Yes	
AA	69/33	**1.0**	Ref.	35/30	**2.5**	1.2, 5.0*	TT	141/79	**1.0**	Ref.	65/85	**2.4**	1.5, 3.8***
AC	142/92	**1.7**	1.0, 2.9	70/80	**2.9**	1.6, 5.1***	TG	107/108	**1.7**	1.1, 2.6*	58/82	**2.3**	1.4, 3.7***
CC	72/97	**3.4**	1.9, 5.6***	44/75	**3.8**	2.2, 6.9***	GG	19/35	**3.4**	1.7, 6.7***	16/18	**2.4**	1.1, 5.3*
	**Preconception paternal smoking**							**Preconception paternal smoking**					
		No			Yes				No			Yes	
AA	56/34	**1.0**	Ref.	50/33	**1.0**	0.5, 2.0	TT	109/77	**1.0**	Ref.	96/92	**1.1**	0.7, 1.9
AC	115/81	**1.3**	0.8, 2.3	99/93	**1.5**	0.9, 2.7*	TG	92/86	**1.2**	0.8, 1.9	73/110	**1.7**	1.1, 2.7*
CC	67/76	**1.9**	1.1, 3.4**	47/103	**3.3**	1.8, 5.9***	GG	19/28	**2.3**	1.2, 4.7*	18/27	**2.0**	1.0, 4.3*
	**Breastfeeding**							**Breastfeeding**					
		No			Yes				No			Yes	
AA	46/35	**1.0**	Ref.	60/32	**0.9**	0.5, 1.8	TT	87/101	**1.0**	Ref.	124/74	**0.6**	0.4, 1.0*
AC	98/88	**1.4**	0.8, 2.6	120/94	**1.4**	0.8, 2.4	TG	71/99	**1.1**	0.7, 1.8	96/103	**1.0**	0.6, 1.5
CC	47/102	**3.0**	1.6, 5.4***	71/83	**2.0**	1.1, 3.6	GG	21/25	**1.1**	0.5, 2.1	16/32	**2.3**	1.1, 4.8*
	**Repeated early common infections** ^**b**^							**Repeated early common infections** ^**b**^					
		No			Yes				No			Yes	
AA	56/40	**1.0**	Ref.	37/24	**0.9**	0.5, 1.9	TT	113/126	**1.0**	Ref.	68/44	**0.6**	0.3, 0.9*
AC	123/111	**1.3**	0.8, 2.3	57/57	**1.6**	0.9, 2.9	TG	97/131	**1.1**	0.7, 1.6*	41/59	**1.1**	0.7, 1.9
CC	67/137	**3.1**	1.8, 5.3***	33/42	**1.7**	0.9, 3.3	GG	22/31	**1.2**	0.6, 2.3	7/20	**3.1**	1.2, 8.0*
	**Birth order**							**Birth order**					
		1			≥2				1			≥2	
AA	35/34	**1.0**	Ref.	71/33	**0.5**	0.3, 1.0*	TT	77/88	**1.0**	Ref.	134/87	**0.5**	0.3, 0.8*
AC	98/93	**1.2**	0.7, 2.2	120/89	**0.8**	0.4, 1.5	TG	74/105	**1.1**	0.7, 1.8	93/97	**0.7**	0.4, 1.1
CC	48/96	**2.7**	1.4, 5.1**	70/89	**1.1**	0.6, 2.1	GG	18/30	**1.7**	0.8, 3.5	19/27	**1.1**	0.5, 2.2

ALL: acute lymphoblastic leukemia; CI: confidence interval; Co: controls; OR: odds ratio; SNP: Single nucleotide polymorphism.

*: *P*<0.05;

**: *P*<0.01;

***: *P*<0.001

^a^ OR and 95% CI estimated by unconditional logistic models including gender and age and parental socio-professional category.

^b^ Restriction to children ≥ 1 year old

The more marked interactions were observed between rs4132601 and maternal insecticide use (IOR = 0.5, 95%CI: 0.3–0.9, *p* = 0.012), breastfeeding (IOR = 1.7, 95%CI: 1.1–2.7, *p* = 0.017) and repeated early common infections (IOR = 2.0, 95%CI: 1.2–3.4, *p* = 0.0070) in the case-control design (Table 3). The magnitudes of the interactions were similar using the case-only design (Table 3).

**Table 3:** Interaction between the SNP rs4132601 (*IKZF1*) and maternal use of home insecticides during pregnancy, preconception paternal smoking, breastfeeding, repeated early common infections and birth order, in their relation with childhood acute lymphoblastic leukemia.

		Case-control						Case only		
		Model without interaction			Model with interaction					
		**OR** ^a^	95%CI	p	**OR** ^a^	95%CI	p	**OR** ^a^	95%CI	p
***IKZF1* (rs4132601)**										
Maternal use of home insecticides during pregnancy (yes vs. no)		**1.6**	1.2, 2.2	**	**2.4**	1.6, 3.7	***			
rs4132601 G allele		**1.4**	1.1, 1.8	**	**1.8**	1.3, 2.4	***			
rs4132601 G allele*maternal use of home insecticides during pregnancy		**-**			**0.5**	0.3, 0.9	*0*.*012*	**0.7**	0.5, 0.9	*0*.*022*
Preconception paternal smoking (yes vs. no)		**1.3**	0.9, 1.7		**1.3**	0.8, 2.0				
rs4132601 G allele		**1.4**	1.1, 1.7	**	**1.4**	1.0, 1.9	*			
rs4132601 G allele* preconception paternal smoking					**1.0**	0.6, 1.5	*0*.*85*	**0.9**	0.7, 1.2	*0*.*61*
Breastfeeding (yes vs. no)		**0.8**	0.6, 1.1		**0.6**	0.4, 0.9	*			
rs4132601 G allele		**1.4**	1.2, 1.7	**	**1.1**	0.8, 1.4				
rs4132601 G allele* breastfeeding					**1.7**	1.1, 2.7	*0*.*017*	**1.3**	1.0, 1.8	*0*.*048*
Repeated early common infections ^b^ (yes vs. no)		**0.8**	0.6, 1.2		**0.5**	0.3, 0.9	*			
rs4132601 G allele		**1.4**	1.1, 1.7	**	**1.1**	0.8, 1.4				
rs4132601 G allele* repeated early common infections					**2.0**	1.2, 3.4	*0*.*0070*	**1.4**	1.0, 1.9	*0*.*061*
Birth order (2+ vs. 1)		**0.6**	0.4, 0.8	***	**0.5**	0.3, 0.8	**			
rs4132601 G allele		**1.4**	1.1, 1.7	**	**1.3**	0.9, 1.7	*			
rs4132601 G allele* birth order					**1.2**	0.7, 1.8	*0*.*53*	**0.9**	0.7, 1.2	*0*.*49*

CI: confidence interval; OR: odds ratio; SNP: Single nucleotide polymorphism.

*:p<0.05;

**: p<0.01;

***: p<0.001

^a^ OR and 95% CI estimated by unconditional logistic models including gender and age and parental socio-professional category.

^b^ Restriction to children ≥ 1 year old

The allelic ORs were only increased among the subjects not exposed to the suspected non-genetic factor, i.e. the children whose mother did not use home insecticides during pregnancy (allelic OR = 1.8, 95%CI: 1.3, 2.4), those who had been breastfed (allelic OR = 1.8, 95%CI: 1.3, 2.5) and those who had had repeated common infections in their first year of life (allelic OR = 2.4, 95%CI: 1.5, 3.8) (Table 4). On the opposite, the relationship between ALL and the G allele of rs4132601 was not present among the subjects exposed to the non-genetic factors, i.e. the children whose mother used home insecticides during pregnancy (allelic OR = 1.0, 95%CI: 0.7, 1.5), those who had not been breastfed (allelic OR = 1.0, 95%CI: 0.8, 1.4) and those who had had no or few common infections in their first year of life (allelic OR = 1.1, 95%CI: 0.8, 1.5) (Table 4). Similar differences in the effect of rs4132601 by the non-genetic factors were observed for the most frequent ALL subtypes, i.e., common B-cell ALL, hyperdiploid common B-cell ALL and ETV6-RUNX1 common B-cell ALL (Table 4).

**Table 4:** Associations between childhood acute lymphoblastic leukemia (ALL) and rs4132601 (*IKZF1*) polymorphisms by non-genetic factors: allelic OR in the whole sample and by ALL major subtypes, in the ESCALE study, France 2003–2004.

		**Maternal use of home insecticides during pregnancy**					**Breastfeeding**					**Repeated early common infections** ^b^				
		No		Yes			Yes		No			Yes		No		
		OR^a^	95%CI	OR^a^	95%CI	p int	OR^a^	95%CI	OR^a^	95%CI	p int	OR^a^	IC95%	OR^a^	95%CI	p int
**Rs4132601 *(IKZF1)*—OR per G allele**																
Whole sample (n = 442 ALL)		**1.8**	1.3, 2.4***	**1.0**	0.7, 1.5	0.012	**1.8**	1.3, 2.5***	**1.0**	0.8, 1.4	0.017	**2.4**	1.5, 3.8***	**1.1**	0.8, 1.5	0.0070
B-com ALL (n = 355)		**1.7**	1.3, 2.4***	**0.9**	0.6, 1.4	0.007	**1.7**	1.2, 2.4**	**1.0**	0.7, 1.4	0.03	**2.3**	1.4, 3.7***	**1.0**	0.8, 1.4	0.0078
Hyperdiploidy B-com ALL (n = 157)		**2.2**	1.4, 3.4***	**1.3**	0.8, 2.2	0.07	**2.2**	1.4, 3.6**	**1.3**	0.8, 2.0	0.10	**3.2**	1.7, 6.0***	**1.3**	0.9, 2.0	0.026
ETV6-RUNX positive B-com ALL (n = 82)		**1.7**	1.0, 2.8	**1.0**	0.5, 1.8	0.18	**1.9**	1.1, 3.5*	**1.0**	0.6, 1.7	0.13	**2.3**	1.0, 5.1*	**1.1**	0.7, 1.7	0.10

ALL: acute lymphoblastic leukemia; B-com ALL: common B-cell ALL; CI: confidence interval; OR: odds ratio; p int: p for interaction

*:p<0.05;

**: p<0.01;

***: p<0.001

^a^ Allelic OR and 95% CI estimated by unconditional logistic models adjusted for gender, age and parental socio-professional category.

^b^ children ≥ 1 year old

Regarding *ARID5B*, the at-risk loci of rs10740055 also interacted with maternal insecticide use, although not significantly. A negative interaction was observed with repeated early common infections (IOR = 0.7, 95%CI: 0.5–0.9, *p* = 0.018) in the case-only design (Table 5).

**Table 5:** Interaction between the SNP rs10740055 (*ARID5B*) and maternal use of home insecticides during pregnancy, preconception paternal smoking, breastfeeding, repeated early common infections and birth order, in their relation with childhood acute lymphoblastic leukemia.

		Case-control						Case only		
		Model without interaction			Model with interaction					
		**OR** ^a^	95%CI	p	**OR** ^a^	95%CI	p	**OR** ^a^	95%CI	p
***ARID5B* (rs10740055)**										
Maternal use of home insecticides during pregnancy (yes vs. no)		**1.6**	1.2, 2.1	**	**2.5**	1.4, 4.5	**			
rs10740055 C allele		**1.6**	1.3, 1.9	***	**1.9**	1.4, 2.4	***			
rs10740055 C allele* Maternal use of home insecticides during pregnancy					**0.7**	0.4, 1.0	*0*.*067*	**0.9**	[0.7–1.2]	*0*.*62*
Preconception paternal smoking (yes vs. no)		**1.3**	1.0, 1.7		**0.9**	0.5, 1.6				
rs10740055 C allele		**1.6**	1.3, 1.9	***	**1.4**	1.0, 1.8	*			
rs10740055 C allele* preconception paternal smoking					**1.3**	0.9, 2.0	*0*.*19*	**1.3**	[0.9–1.7]	*0*.*11*
Breastfeeding (yes vs. no)		**0.8**	0.6, 1.1		**1.0**	0.6, 1.8				
rs10740055 C allele		**1.6**	1.3, 2.0	***	**1.8**	1.3, 2.4	***			
rs10740055 C allele* breastfeeding					**0.8**	0.5, 1.2	*0*.*36*	**0.9**	[0.7–1.1]	*0*.*28*
Repeated early common infections ^b^ (yes vs. no)		**0.9**	0.6, 1.2		**1.3**	0.7, 2.3				
rs10740055 C allele		**1.6**	1.3, 2.0	***	**1.8**	1.4, 2.4	***			
rs10740055 C allele* repeated early common infections					**0.7**	0.5, 1.1	*0*.*17*	**0.7**	[0.5–0.9]	*0*.*018*
Birth order (2+ vs. 1)		**0.5**	0.4, 0.7	***	**0.6**	0.4, 1.1				
rs10740055 C allele		**1.6**	1.3, 1.9	***	**1.7**	1.3, 2.3	***			
rs10740055 C allele* birth order					**0.9**	0.6, 1.3	*0*.*51*	**1.0**	[0.8–1.3]	*0*.*94*

CI: confidence interval; OR: odds ratio; SNP: Single nucleotide polymorphism.

^a^ OR and 95% CI estimated by unconditional logistic models including gender and age and parental socio-professional category.

^b^ Restriction to children ≥ 1 year old

*: p<0.05;

**: p<0.01;

***: p<0.001

However, the tests were no longer significant when the Bonferroni correction for multiple testing was applied (10 tests for 2 SNPs and 5 non-genetic factors; corrected alpha risk = 0.005).

## Discussion

In recent years some *IKZF1* and *ARID5B* SNPs have been shown to be clearly associated with childhood ALL. The present study showed that the at-risk allele of *IKZF1* interacted negatively with maternal use of home insecticides during pregnancy, and positively with factors related to early immune modulation, breastfeeding and history of repeated early common infections. There was no interaction with preconception paternal smoking.

However, chance findings cannot be ruled out as the study was exploratory with respect to the interaction between the at-risk alleles and the non-genetic suspected risk factors. We have limited multiple testing by focusing only on the 2 loci the most significantly associated with ALL in the present study and the literature [4–12], and on the 5 non-genetic suspected ALL risk factors that were of sufficient prevalence in the ESCALE case-control study. Therefore, the other ALL risk loci identified by the GWAS, in *CEBPE*, *CDKN2A*, *GATA3* and *PIP4K2A*, were not investigated. Larger consortium-based studies are needed to comprehensively investigate the interactions between each of the ALL susceptibility polymorphisms and non-genetic suspected risk factors.

There was a substantial number of losses in the present study, related to non-participation, refusal in providing genetic material, and insufficient amount of DNA, leading to the potential for selection bias. Employment in the most qualified category ‘Intellectual and scientific jobs, managers and intermediate professions’ was significantly more frequent in the parents of the genotyped controls (56%) than in those of the other non-genotyped Caucasian controls (39%), but significantly less frequent in the parents of the genotyped cases (34%) than in those of the other non-genotyped Caucasian cases (45%). However, the frequency of the at risk alleles of rs4132601 and rs10740055, differed only slightly according to whether the parents’ belonged to the most qualified professional category, among both the controls and the cases. This, and the fact that the associations of ALL with the non-genetic factors were similar in the genotyped and non-genotyped children, suggest no or little effect of the loss of subjects on the estimation of the interactions.

The cases and controls were genotyped separately using different methods, but the genotypes obtained with the pangenomic and customized BeadChips were shown to be fully concordant in a sample of 96 cases genotyped using both platforms [6]. Past exposures may not be remembered perfectly by the mothers and some misclassifications inherent in the retrospective design of the study could not be prevented. The potential for recall bias is likely to be greater for a history of common infections and the use of pesticides during pregnancy, which are sporadic events, than for breastfeeding, paternal smoking or birth order. In particular, underreporting of medically diagnosed infections has been reported [35–37], and, in the United Kingdom Childhood Cancer Study, an inverse association was observed between ALL and a history of clinically diagnosed infections as reported by the mothers, but a positive association was observed when relying on information from medical records [37,38]. In addition, studies based on medical records or health claims databases reported null [39,40] or positive associations [41–43] between a history of clinically diagnosed infection and ALL, suggesting that children who develop leukemia are more likely to have had clinically diagnosed infections in infancy. The present study reported an inverse association between ALL and maternal report of repeated common infections, medically diagnosed or not, considered as a surrogate for frequent exposure to infectious agents. The influence of case/control status on that recall, and the direction of the bias remain difficult to predict. However, with regard to the specific goal of the present analyses, the exposure misclassifications are not expected to differ according to the polymorphisms of interest and are not likely to have resulted in strong bias in the estimates of the interactions.

To our knowledge, the interactions between the genetic variants identified by the GWAS and some non-genetic factors have been investigated in only two previous studies. Linabery et al. reported no interaction between some *ARID5B* and *IKZF1* SNPs and some selected demographic factors, birth weight and maternal age, using a case-only design [44]. In a recent Australian study, there was an evidence of interaction between rs4132601 risk genotype at *IKZF1* and paternal preconception smoking (p = 0.05) and maternal use of folic acid (p = 0.04) [12]. In the present study, we did not observe an interaction with paternal smoking, and the power was too limited for investigating interaction with folic acid supplementation, whose prevalence was low in France during the study period [45]. Interestingly, in the present study and in the Australian study, the increase in ALL risk in carriers of the G allele of *IKZF1* rs4132601 was not observed in the event of exposure to the suspected risk factors (or in the absence of exposure to the suspected protective factor). Biologically plausible explanations for our observations are lacking and further studies are needed to replicate (or not replicate) our observations. One possible explanation may be the modification of *IKZF1* expression by the environment, as growing evidence suggests that environmental factors, including pesticides and breastfeeding, may modify the expression of genes involved in key cell functions, in particular through epigenetic deregulation [20–27]. To our knowledge, no experimental study has yet investigated the possibility that exposure to pesticides or a lack of early immune stimulation may reduce the expression of *IKZF1* or other genes involved in lymphoid differentiation, or that the impact of the non-genetic factors may differ depending on the gene polymorphisms. The decrease in the expression of one or several genes involved in a given cell function, following exposure to an environmental factor, might possibly result in the same effect as carrying a specific polymorphism associated with lower expression of the genes, potentially leading to a negative interaction. Statistical interactions on a multiplicative scale might also be observed if environmental factors and genetic factors would lead to leukemia through different tumorigenic pathways.

In conclusion, this study suggests the possibility of a negative interaction between ALL susceptibility locus in *IKZF1* and prenatal insecticide exposure, and of positive interactions between the at-risk allele and breastfeeding and history of repeated early common infections. Further studies are needed to evaluate whether these observations are chance findings or reflect true underlying mechanisms.

## Supporting Information

S1 Fig Diagram of the procedure used to select population controls in the ESCALE study, France, 2003–2004. (TIF)

S1 Table ESCALE sampling and final inclusions, France 2003–2004. (DOC)
