# Supplementary figures and images for: ARID5B, IKZF1 and Non-Genetic Factors in the Etiology of Childhood Acute Lymphoblastic Leukemia: The ESCALE Study
Source: PLoS One. 2015 Mar 25;10(3):e0121348. doi: 10.1371/journal.pone.0121348 (PMC4373901; doi:10.1371/journal.pone.0121348)

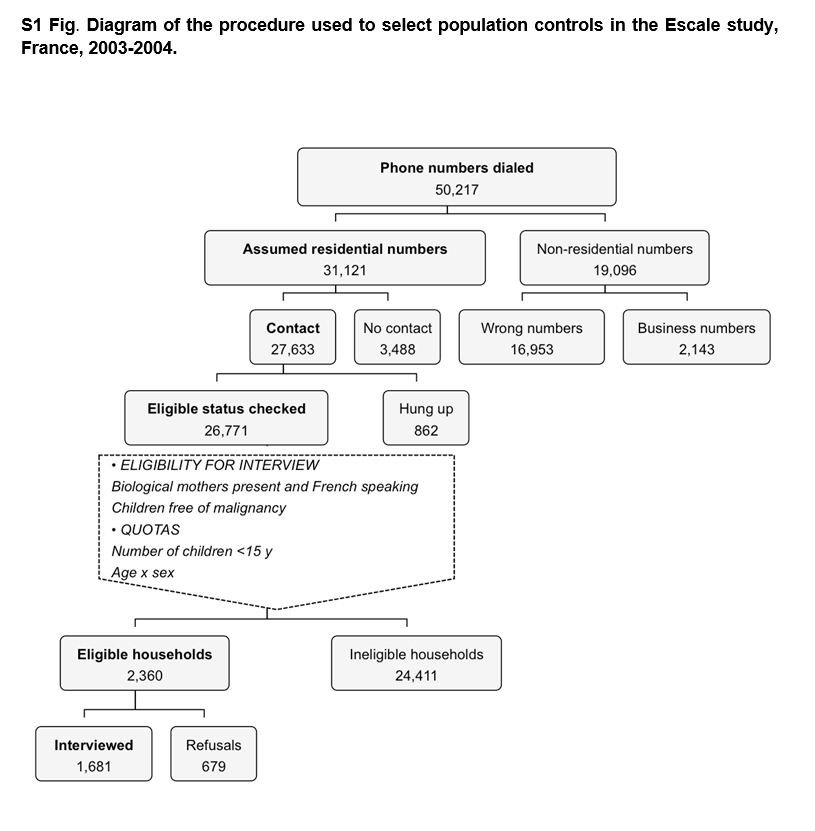

Supplement: S1 Fig — (TIF) [file pone.0121348.s001.tif]
