# Supplementary material for: ARID5B, IKZF1 and Non-Genetic Factors in the Etiology of Childhood Acute Lymphoblastic Leukemia: The ESCALE Study
Source: PLoS One. 2015 Mar 25;10(3):e0121348. doi: 10.1371/journal.pone.0121348 (PMC4373901; doi:10.1371/journal.pone.0121348)
Supplement: S1 Table — (DOC) [file pone.0121348.s002.doc]

**S1 Table. ESCALE sampling and final inclusions, France 2003-2004.**

| **Selection criteria** |  | **ALL** | **Controls** |
| --- | --- | --- | --- |
| **ESCALE selection** | Eligibility | 714 | 2360 |
|  | Inclusion (interviews) | 648 | 1681 |
| **DNA and genotyping** | Biological specimen **^a^** | 619 | 810 |
|  | Successful genotyping (sufficient data) | 513 | 570 |
|  | Successful quality control | 477 | 461 |
| **Additional selections** | Exclusion of Down's syndrome | 471 | 461 |
|  | European-descent (≥2 grandparents born in Europe) | 434 | 442 |
| **ALL immunological subtypes** | Pro-B ALL | 10 |  |
|  | Common B-cell ALL | 355 |  |
|  | Burkitt cell ALL | 19 |  |
|  | T cell ALL | 40 |  |
|  | Others or unclassified | 10 |  |
| **ALL ploidy** | Normal karyotype | 103 |  |
|  | <46 chromosomes | 17 |  |
|  | Pseudodiploidy | 95 |  |
|  | 47-50 chromosomes | 44 |  |
|  | >50 chromosomes | 125 |  |
|  | Karyotype failure | 50 |  |
| **ALL molecular markers** | ETV6-RUNX | 83 |  |
|  | MLL | 7 |  |
|  | MLL or 11q23 abnormalities | 11 |  |
|  | BCR-ABL1 | 7 |  |

ALL: acute lymphoblastic leukemia;

**^a^**Blood sample for the cases and buccal swab brushes for the ESCALE controls
